# Regulation of urinary bladder function by protein kinase C in physiology and pathophysiology

**DOI:** 10.1186/s12894-015-0106-6

**Published:** 2015-11-04

**Authors:** Joseph A. Hypolite, Anna P. Malykhina

**Affiliations:** Division of Urology, Department of Surgery, University of Colorado Denver, Anschutz Medical Campus, 12700 E 19th Ave. Mail Stop C317, Aurora, CO 80045 USA

**Keywords:** Detrusor smooth muscle, Micturition, Nerves, Contractility, Signaling pathways, Bladder pathophysiology

## Abstract

**Background:**

Protein kinase C (PKC) is expressed in many tissues and organs including the urinary bladder, however, its role in bladder physiology and pathophysiology is still evolving. The aim of this review was to evaluate available evidence on the involvement of PKC in regulation of detrusor contractility, muscle tone of the bladder wall, spontaneous contractile activity and bladder function under physiological and pathophysiological conditions.

**Methods:**

This is a non-systematic review of the published literature which summarizes the available animal and human data on the role of PKC signaling in the urinary bladder under different physiological and pathophysiological conditions. A wide PubMed search was performed including the combination of the following keywords: “urinary bladder”, “PKC”, “detrusor contractility”, “bladder smooth muscle”, “detrusor relaxation”, “peak force”, “detrusor underactivity”, “partial bladder outlet obstruction”, “voltage-gated channels”, “bladder nerves”, “PKC inhibitors”, “PKC activators”. Retrieved articles were individually screened for the relevance to the topic of this review with 91 citations being selected and included in the data analysis.

**Discussion:**

Urinary bladder function includes the ability to store urine at low intravesical pressure followed by a subsequent release of bladder contents due to a rapid phasic contraction that is maintained long enough to ensure complete emptying. This review summarizes the current concepts regarding the potential contribution of PKC to contractility, physiological voiding, and related signaling mechanisms involved in the control of both the storage and emptying phases of the micturition cycle, and in dysfunctional voiding. Previous studies linked PKC activation exclusively with an increase in generation of the peak force of smooth muscle contraction, and maximum force generation in the lower urinary tract. More recent data suggests that PKC presents a broader range of effects on urinary bladder function including regulation of storage, emptying, excitability of the detrusor, and bladder innervation.

**Summary:**

In this review, we evaluated the mechanisms of peripheral and local regulation of PKC signaling in the urinary bladder, and their impact on different phases of the micturition cycle under physiological and pathophysiological conditions.

## Background

Physiological contractions of the detrusor smooth muscle (DSM) involve the ability to generate and maintain contractile force during the emptying phase of the micturition cycle [1–13]. Initial animal studies suggested that PKC plays a minimal role in DSM contractility [14–19]. This conclusion was based on the failure of PKC to make a significant contribution to maximal force generation in response to muscarinic receptor agonists [14, 16, 18]. Subsequent investigations provided evidence that PKC likely has dual effects on bladder function regulating both contractility and relaxation of the detrusor [3, 7, 8, 20, 21]. For instance, it was determined that low levels of PKC stimulation could inhibit spontaneous contractions and lower basal resting tone of DSM *in vitro*, thereby, likely contributing to bladder storage [8], while high levels of PKC activity contributed to an increase and maintenance of total DSM force (integral force) required for bladder emptying [22].

**Table 1:** Effects of PKC signaling on bladder function

PKC Drugs	Tissue	Methods	Physiological condition	Species	Effect on bladder function	Reference
PBBu	Muscle strips	*In vitro*	Normal	Rabbit, Rat	Inhibition of spontaneous contraction	[8]
(PKC activator, 1nM–50nM)						[22]
PDBu	Muscle strips	*In vitro*	Normal	Rabbit	Increased contractility	[8, 25, 28]
(100nM-1 μM)						
PDBu	Muscle strips	*In vitro*	Normal	Rat	Increased nerve sensitivity, force maintenance	[8, 22, 31]
(100nM-1 μM)						
PDBu	Bladder	*In vivo* (cystometry)	Normal	Rat	Bladder emptying, and frequency of urination	[22]
(1 μM)						
PDBu	Muscle strip	*In vitro*	Normal	Rabbit	Activation of ROCK, Increased DSM tone	[28]
PDBu	Muscle strips	*In vitro*, EFS	Normal	Rat	Increased peak force and area; increased release of acetylcholine.	[30]
PDBu	Bladder myocytes	*In vitro*	Normal	Mice	Increased Cav1.2 current	[33]
PDBu	α-toxin muscle strips	*In vitro*	Normal	Guinea pig	Increased Ca^2+^ sensitivity	[80]
PDBu	Muscle strips	*In vitro*	Obese	Mice	Increased contractility	[26]
(0.001–3 μM)			PBOO			[60]
PDBu	Muscle strips	*In vitro*	PBOO	Rabbit	Decreased contraction and PKC activity	[25, 27]
PMA	DSM cells & Muscle strips	*In vitro*,	Normal	Guinea pig	Inhibition of K_ATP_ and BK currents; increased DSM contraction	[22, 31, 47]
(PKC activator)		Patch-clamp recordings				
Bim-1	Muscle strips	*In vitro*	Normal	Rabbit	Increased spontaneous contractions; decreased force maintenance; decreased peak force; decreased void; increased non-voiding contractions	[8, 22, 24]
(PKC inhibitor)	Bladder	*In vivo*	Normal	Rat		
Ro318220	Muscle strips	*In vitro*	Normal	Rat	Inhibition of force maintenance;	[22]
(PKC inhibitor)						
	Bladder	*In vivo*	Normal	Rat	Increased non-voiding contractions; decreased void volume	
GF109203X	Muscle strips	*In vitro*	Normal	Guinea pig; rat	Inhibition of Ca^2+^ sensitization	[17]
(PKC inhibitor)						
Carbachol	DSM cells	*In vitro*, Patch-clamp recordings	Unknown	Human	Inhibition of BK channels	[76]
Carbachol	DSM	*In vitro*	Unknown	Human	Increased Ca++ sensitivity of contractile filaments	[68]

Stimulation of muscarinic receptors triggers activation of phospholipase C (PLC)/PKC downstream signaling associated with smooth muscle contractility via G-protein-coupled receptors [15, 16, 23, 24]. Animal studies confirmed that the same pathway is present in the urinary bladder [15], and that PKC is involved in a variety of cellular events that regulate DSM contractility. Among these are the effects on the smooth muscle itself [3, 7, 8, 17, 25–30], expression and function of ion channels [8, 26, 31–33], and intrinsic bladder nerves assessed by *in vitro* contractility studies on isolated DSM strips during electric field stimulation (EFS) [22, 34, 35]. In this review, we summarized current data on the involvement of PKC signaling in modulation of bladder function with focus on the contractile force of DSM, relaxation of the muscle during filling phase, as well as the ability of the detrusor to develop and maintain muscle tone throughout the micturition cycle under physiological and pathophysiological conditions.

### Methods

This is a non-systematic review of the published literature which summarizes the available animal and human data on the role of PKC signaling in the urinary bladder under different physiological and pathophysiological conditions. A wide PubMed search was performed including the combination of the following keywords: “urinary bladder”, “PKC”, “detrusor contractility”, “bladder smooth muscle”, “detrusor relaxation”, “peak force”, “detrusor underactivity”, “partial bladder outlet obstruction”, “voltage-gated channels”, “bladder nerves”, “PKC inhibitors”, “PKC activators”. Retrieved articles were individually screened for the relevance to the topic of this review with 91 citations being selected and included in the analysis.

Table 1 summarizes the effects of a variety of PKC inhibitors, activators, and cholinergic agonists on PKC signaling and contractility of DSM.

## Discussion

The aim of this review was to evaluate the available evidence on the involvement of PKC in regulation of detrusor contractility, muscle tone of the bladder wall, spontaneous contractile activity and bladder function under physiological and pathophysiological conditions.

### Modulation of DSM excitability and ion channel activity by PKC

Bladder smooth muscle cells express a variety of ion channels controlling cell excitability. Voltage-gated calcium channels (VGCC), large- (BK), and small- (SK) conductance Ca^2+^-activated potassium channels, and ATP-sensitive potassium channels (K_ATP_) are the main channels involved in the control of detrusor excitability and excitation-contraction coupling [4, 15, 26, 32, 36].

#### Voltage gated calcium channels

**Fig. 1:**
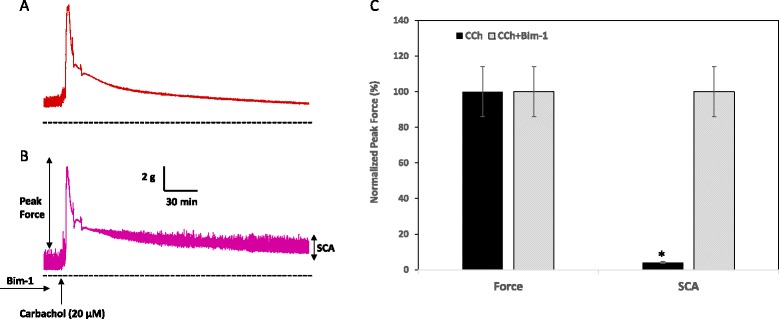
Effects of carbachol (CCh) on the contractile force and spontaneous contractions in DSM *in vitro.* Isolated muscle strips from rabbit bladders were mounted in organ baths with Tyrode’s buffer (equilibrated with 95%O_2_/5%CO_2_) and allowed to develop spontaneous contractions. The muscle strip shown in **a** was treated with 20 μM of carbachol while the muscle strip in **b** was first pre-incubated with Bim-1 (28 nM), a PKC inhibitor, for 45 min prior to adding carbachol. Treatment with carbachol increased peak muscle force and reduced the amplitude of spontaneous contraction (SCA) by 96 ± 14 %, (*n* = 4, *p* < 0.05 in **a**), however, carbachol had no effect on spontaneous contraction amplitude (SCA) after PKC inhibition with Bim-1 (**b**). **c** Normalized amplitude of the peak force of the contractile response to CCh. Maximal amplitude was taken as 100 %. Light bars show the effects of carbachol on peak force and SCA without Bim-1. Dark bars show the effect of carbachol on peak force and SCA with Bim-1 application

Several types of VGCC are expressed in the detrusor [15] with L-type VGCC being the main ion channel responsible for the onset of action potentials in DSM cell, as well as for the contractile response of the detrusor to muscarinic receptor stimulation [37–42]. VGCC have also been shown to mediate spontaneous contractions in DSM cells [4]. Recent studies determined that one of the PKC activators, phorbol-12,13-dibutyrate (PDBu), inhibited voltage-dependent spontaneous contractions in isolated DSM strips from rabbit and rat bladders at low concentrations [8, 22]. Additionally, PKC activation by PDBu inhibited receptor-induced phasic activity in the bladders of newborn mice but not in adult bladders [29]. Inhibition of DSM spontaneous contractility by PKC activator, PDBu, was also observed in isolated muscle strips of the human DSM (unpublished observations from our laboratory) and rat DSM [22]. Utilizing whole-cell patch clamp technique, Kajioka et al. (2002) demonstrated an increase in the amplitude and current density of L-type Ca^2+^ channels upon depolarization in pig and human bladders [43]. Interestingly, in the same study, carbachol significantly diminished the amplitude of VGCC current which was sensitive to nifedipine. The mechanisms of the opposing actions of carbachol on VGCC are not entirely clear. It is well established that initial phasic contraction of DSM in response to carbachol stimulation is associated with membrane depolarization and opening of VGCC [15]. However, this initial massive increase in intracellular calcium is followed by subsequent closing of VGCC and a decrease in intracellular Ca^2+^ due to its storage in the intracellular stores [44]. These subsequent changes are associated with a quiescent state of the muscle after initial phasic contraction, and, likely, underlie the absence of spontaneous contractile activity during relaxation phase after muscarinic stimulation (Fig. 1). This action may be mediated by carbachol-induced activation of PKC [24] at low or resting calcium concentration, since PKC stimulation by PDBu was reported to activate BK channels resulting in inhibition of spontaneous contractions [8, 22]. Interestingly, pre-incubation of the DSM strips *in vitro* with the PKC inhibitor, Bim-1, before carbachol stimulation preserved spontaneous contractility during relaxation phase (Fig. 1).

#### Effects of PKC activation on BK channels

The storage phase of the micturition cycle requires a quiescent smooth muscle that can accommodate increasing volumes at low intravesical pressure. Potassium channels contribute to this process by helping maintain the resting membrane potential via regulation of both the intracellular calcium concentration, and calcium entry into the cell from extracellular sources [32, 36, 45]. Several types of potassium channels are expressed in DSM, and are known to be involved in this regulation. Among them, BK and K_ATP_ channels were shown to be regulated by PKC. PKC was shown to be involved in the regulation of BK channels expressed in DSM tissue of rabbit [8] and guinea pig [31] bladders. Hypolite et al. (2013) reported that low levels of PKC stimulation by the PKC activator, PDBu, inhibited spontaneous myogenic contractions, and reduced basal DSM tone in rabbit DSM, which was dependent on activation of BK channels [8]. Additionally, PDBu was unable to inhibit spontaneous contractions in the presence of the BK channel blocker, iberiotoxin, suggesting that the mechanism of PDBu-induced inhibition of spontaneous contractions was via activation of BK channels. Utilizing whole-cell patch clamp recordings in guinea pig DSM cells, Hristov et al. (2014) confirmed that PKC does regulate BK channel activity, however, these authors reported that PKC stimulation with PMA increased muscle force along with amplitude of spontaneous contractions [31]. The variability in the responses to PKC stimulation reported by the abovementioned studies could be attributed to different species (guinea pigs *vs* rabbits), or the use of distinct PKC activators (PDBu versus PMA), as well as slightly different methodological approaches.

#### Modulation of K_ATP_ channels by PKC

**Fig. 2:**
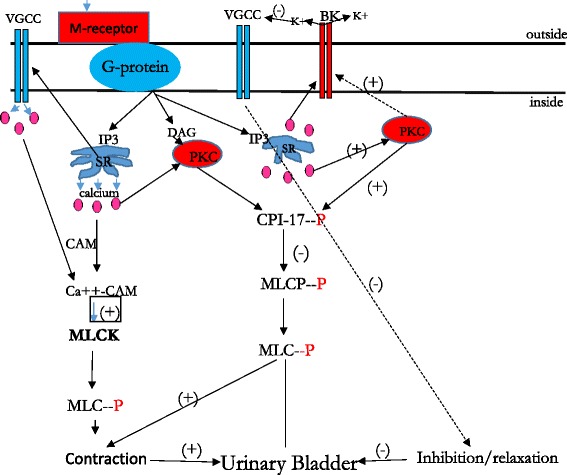
Schematic presentation of the PKC signaling pathways involved in the regulation of urinary bladder function. Abbreviations are spelled out in the text of the manuscript. (+) Activation of the pathways, (−) means Inhibition, (P) is Phosphorylation. Broken arrows indicate potential pathways for PKC-induced inhibition of DSM tone via activation of BK channels

Similar to BK channels, K_ATP_ channels also participate in the control of detrusor excitability across a broad spectrum of mammalian species including guinea pigs [46, 47], humans [48], and rats [49]. Activation of these channels induces DSM relaxation in response to K_ATP_ channel openers pinacidil [50], and cromakalim [51]. Activation of M_3_ receptors can induce inhibition of these channels in smooth muscle cells via PKC signaling pathways [52]. Stimulation of muscarinic receptors by carbachol was shown to inhibit K_ATP_ currents by 60.7 %, while activators of PKC inhibited K_ATP_ channels by 74 % [52]. Additionally, PKC blockers used before stimulation with muscarinic receptor agonists, significantly reduced carbachol-induced inhibition of K_ATP_ currents confirming that muscarinic-dependent inhibition of K_ATP_ currents is mediated via PKC pathways [52]. This speculation is consistent with *in vitro* studies showing that the PKC inhibitor, Bim-1, reduced both intrinsic basal tone, and maintained force [8], while awake cystometry performed in rats revealed that inhibition of PKC resulted in increased frequency of urination, and decreased void volume [22]. Schematic presentation of the ion channels involved in regulation of BSM excitability and contractility as well as downstream signaling including PKC pathways is depicted in Fig. 2.

### The link between muscarinic receptors and PKC signaling in the control of detrusor contractility

Muscarinic receptor type 3 (M_3_) signaling via G_q/11_ G-protein-coupled receptors includes activation of PLC/PKC as determined for several types of smooth muscle [23] including the DSM of the bladder [16, 53–55]. Initial experiments in the urinary bladder established that inhibition of PLC/PKC pathway by a number of specific inhibitors had no significant effect on maximal contractility of rat bladder muscle strips in response to carbachol stimulation leading to the conclusion that PLC/PKC-dependent signaling via M_3_ receptors does not significantly contribute to DSM contractility [16]. This data was further buttressed by the studies showing that direct inhibition of PKC had no significant effect on the peak contractile force in response to both carbachol and EFS stimulation [8, 14].

An exception to the above findings was the observation that one of the PKC inhibitors, Bim-1, did reduce maximal force in response to carbachol upon application of a higher concentration of the inhibitor [24]. Another PKC inhibitor, GF 109203X (1 μM), was also reported to reduce peak force in control bladders by 29 % [56]. Subsequent studies clarified that inhibition of PKC with Bim-1, at lower concentrations, did not significantly affect electric field stimulation (EFS)-induced maximal amplitude of DSM contractions, however, significantly reduced the integral, or total force [8, 22]. The integral (total) force is calculated as the area under the curve of a single recorded contraction, and represents the ability of DSM to maintain muscle force required for physiological bladder emptying [8]. The effects of Bim-1 observed *in vitro* were further confirmed *in vivo* by cystometric recordings in unanesthetized rats [22]. During urodynamic recordings (cystometry), the urinary bladder was slowly infused with a solution containing Bim-1, causing the voided urine volumes to decline progressively in comparison with infusion of saline in the control group. These *in vivo* results supported the importance of PKC for muscle force maintenance associated with bladder emptying.

Additional studies confirmed the involvement of PKC in the maintenance of muscle force at the molecular and biochemical level [24]. Stimulation of muscarinic receptors in the smooth muscle results in inhibition of myosin light chain (MLC) phosphatase activity, an increase in MLC phosphorylation and, therefore, contractile force. One of the underlying pathways for inhibition of MLC phosphatase activity is protein kinase C (PKC)-catalyzed phosphorylation of CPI-17 protein [57, 58]. Wang and co-authors evaluated phosphorylation of protein complex Thr(38)-CPI-17, the downstream target of PKC signaling, and established that PKC activation increased Thr(38)-CPI-17 phosphorylation throughout the phasic and tonic portions of carbachol stimulation confirming that PKC is involved in maintaining the contractile force in the detrusor [24].

Limited contribution of the PLC/PKC pathway to the peak amplitude of the contractile response in DSM seems to be physiologically justified as the maximal contractile force of smooth muscle is predominantly controlled by calcium/calmodulin-dependent activation of myosin light chain kinase (MLCK) followed by subsequent phosphorylation of the myosin light chain (MLC) [59]. Since stimulation of DSM by muscarinic receptor agonists leads to a rapid rise in intracellular calcium followed by an equally rapid decline of the calcium to almost basal levels [44], it is highly likely that PKC-induced calcium sensitization via Gq_11_ receptors [3] plays a major role in the maintenance phase of the contraction that is critical for bladder emptying. This mechanism may partly explain why inhibition of PKC significantly reduces DSM force maintenance, and bladder emptying, without significantly affecting peak force generation [14, 22].

### Role of PKC in regulation of spontaneous contractions in the urinary bladder

Protein kinase C is also involved in other aspects of DSM contractility, including the inhibition of basal DSM tone, and spontaneous myogenic contractions [8, 22]. For instance, PDBu, a PKC activator, inhibits spontaneous contractions, and basal myogenic tone at low levels of application (1–50 nM) whereas high concentrations of the drug increase the sensitivity to EFS stimulation *in vitro*, and also trigger micturition contractions *in vivo* confirming a dual, concentration-dependent activation profile of PKC effects in DSM [22]. The ability of PKC to modulate basal DSM tone, and inhibit spontaneous contractions at low levels of stimulation may have physiological implications for bladder storage function, while its contribution to the maintenance of DSM force and nerve-mediated contractions may be important for bladder emptying.

Spontaneous myogenic contractions of DSM have been recorded *in vivo* and *in vitro* in several species including rabbit [8, 11], rat [60], guinea pig [31], pig [40], and humans [61]. The mechanism of these contractions involves calcium entry into the smooth muscle cells via VGCC [40, 43, 62, 63] to trigger action potential generation, while the repolarization phase is thought to be mediated mainly by opening of calcium-activated potassium channels and exit of potassium ions from the smooth muscle cells leading to muscle relaxation [64]. Early reports suggested that spontaneous large-amplitude contractions recorded *in vitro* in isolated DSM strips were likely an artifact with no significant functional role [11]. Subsequent studies suggested that these contractions participate in the maintenance of an intrinsic DSM tone by helping the smooth muscle adjust its length and shape in response to bladder filling [4]. They have also been reported to be associated with peripheral sensory processing that informs the need to void as the bladder approaches capacity [6].

The inhibitory effect of the PKC activator, PDBu, on spontaneous contractions was linked to activation of BK channels, subsequent hyperpolarization of the membrane, and restriction of calcium entry via VGCC [8, 65]. Freshly isolated DSM strips from the rabbit bladder exhibit high amplitude spontaneous contractions which decline over several hours, however, the amplitude of contractions remains at a high level in the presence of PKC inhibitor Bim-1 [8]. This observation suggests that endogenous PKC signaling likely maintains the amplitude of spontaneous contractions at a low, physiological level under normal physiological conditions. This assumption is supported by the fact that commercially available PKC activator, PDBu, at low levels of stimulation (1–50 nM), accelerated the decline in amplitude of spontaneous contractions over time *in vitro* [8]. PDBu was also reported to inhibit carbachol-induced phasic contractions in neonatal bladders associated with the effects on T-type channels [29], and was also observed to inhibit spontaneous contractions in rat DSM [22].

### Effects of calcium on PKC activity

Protein kinase C conveys both calcium-dependent [66–68], and calcium-independent [3, 68, 69] effects on DSM contractility *in vitro* mediated via inhibition of MLCP. It is still unknown if these separate effects are mediated by different PKC isoforms. However, the ability of PKC to mediate both of these actions in DSM may be well suited to urinary bladder storage and emptying function. The storage phase requires the maintenance of basal intrinsic DSM tone at low calcium concentrations which can be mediated, in part, by calcium-independent contractile effects of PKC involved in an increase in tone during the storage phase. This process may be aided by the stretch of the bladder wall associated with an up-regulation of the proteins involved in calcium sensitization [70] and contributing to the basal tone during the early phase of the micturition cycle. However, as the bladder continues to expand, it is likely that enhanced stretch-induced calcium release [71], and calcium-dependent PKC activity increase wall tension further as the bladder approaches capacity. This dual, calcium-independent and dependent, flexibility of PKC may be vital in helping ensure that wall tension rises in a controlled fashion so as to prevent steep increases in bladder pressure during the storage phase of the micturition cycle. PKC-dependent effects have been observed in DSM in response to EFS, which activates the intramural nerves, suggesting that PKC-dependent regulation of neuronal function in DSM is also a distinct possibility [65, 72–74].

### PKC signaling in the human bladder

In comparison to extensive animal data, very little information is available regarding the effects of PKC on human bladder contractility and relaxation. Early studies using human tissues suggested that PKC did not significantly contribute to human bladder contractions [14]. However, only the peak amplitude of contraction (maximum force) was evaluated in the presence of various PKC inhibitors, and no difference was found in peak force generation in comparison to the absence of inhibitors. Since bladder emptying requires both, the generation of peak force and the ability to maintain force, it is possible that PKC could play a more significant role in force maintenance in the human DSM in addition to force generation.

A recent study [75] provided evidence that carbachol, a muscarinic receptor agonist, could indirectly inhibit large conductance Ca^2+^-activated potassium (BK) channels in human DSM cells leading to increased excitability [76]. This is an interesting finding as the ongoing studies in our laboratory using human DSM indicate that carbachol can induce a significant increase in phasic contractions in some human DSM isolated strips, but not in all of them (unpublished data). It is possible that PKC could be an important secondary messenger between muscarinic receptors and BK channels in the human detrusor. Therefore, further studies are warranted to comprehensively characterize the role of PKC in the regulation of cholinergic activity in the human DSM under normal and pathologic conditions.

### Regulation of bladder function by PKC under pathophysiological conditions

The role of PKC in bladder pathophysiology is largely related to DSM contractile dysfunction and dysfunctional voiding associated with partial bladder outlet obstruction (PBOO), and detrusor overactivity (DO). Initial studies, using a PBOO model in rabbits, reported that PKC signaling under pathophysiological conditions was uncoupled from its downstream targets and produced little or no force in response to PKC activator, PDBu [27]. Chang et al. [25] also determined that decreased force generation in decompensated PBOO bladders in response to PKC activator, PDBu, was associated with reduced expression of PKC and increased frequency of urination in the obstructed bladders. It should be noted, however, that a broad range of contractile and metabolic dysfunctions were linked to decompensated bladder function in PBOO models [77, 78]. Further studies confirmed a deficit in the PKC pathway in guinea pig DSM after PBOO [79]. Partial bladder outlet obstruction causes two phenotypes of bladder dysfunction referred to as compensated and decompensated bladders [25, 77]. Compensated bladders are characterized mainly by increased smooth muscle hypertrophy, however, residual urine and frequency of micturition have been reported in compensated bladders associated with PKC dysfunction [25, 80, 81]. Decompensated bladders develop an increase in extracellular matrix including collagen and connective tissue resulting in a loss of bladder compliance, decreased contractility, and a substantial loss of bladder emptying function [25, 77, 78, 80–83]. Changes in PKC expression and activity in a PBOO model seem to be dependent on the species and degree of obstruction. Thus, in the rabbit model of PBOO, decompensated bladders revealed a reduction in PKC expression, activity and force generation associated with reduced bladder emptying and frequency of urination, while compensated bladders exhibited increased PKC expression [25]. However, in the rat bladders with PBOO, PKC expression was reduced in both compensated, and decompensated bladders [81]. Additionally, recent *in vivo* studies established that inhibition of PKC during cystometric recordings in awake rats induced bladder decompensation characterized by an increase in non-voiding contractions (DO), and a significant decrease in void volume [22].

Increased PKC-mediated frequency of urination along with DO have also been reported in the absence of PBOO. In a model of obesity associated with insulin resistance, PKC expression was significantly higher in the bladders of obese mice [26] which showed increased contractility to PDBu, increased frequency, and non-voiding contractions. The changes in the voiding cycle were reversed by metformin treatment which restored high PKC expression in the urinary bladders. High levels of PKC stimulation in rat bladders [22], and high level of expression in compensated rabbit bladders [25] were associated with enhanced nerve-mediated contractions, and frequency of urination, respectively. Thus, a high level of PKC activity and expression may lower the threshold for activation of intramural nerves leading to neurogenic frequency of urination in both diabetic, and compensated bladders. It was established that the protein adiponectin, expressed in adipose tissue, contributed to the increased expression of calcium-dependent PKC-α and enhanced contractile force of DSM in adiponectin-sense transgenic mice [67]. Additionally, carbachol-induced phosphorylation of PKC-α was also elevated in these animals in comparison with WT mice suggesting that adiponectin increases calcium dependency of DSM contractions mediated by PKC-α expression.

It was previously shown that reduced expression of PKC in decompensated rabbit bladders was associated with increased frequency, decreased void, and consequently large residual volumes [25]. Additional cystometric studies in awake rats also revealed that pharmacological inhibition of PKC by Bim-1, and Ro318220 induced an increase in non-voiding contractions, frequency, and decreased void volume [22]. Interestingly, both inhibitors of PKC significantly reduced the ability to maintain muscle force of DSM but did not affect the sensitivity of force generation in response to EFS-mediated nerve stimulation. These data indicate that resting and low levels of PKC activity do not have a significant effect on nerve-mediated contractions in DSM. The ability to maintain DSM force is largely a function of the smooth muscle, and involves calcium sensitization mechanisms mediated by PKC, and Rho-kinase in a calcium-independent manner [3, 8, 24, 68]. Therefore, the frequency of micturition induced by reduced expression of PKC in decompensated rabbit bladders, and pharmacologic inhibition during cystometry in rat bladders are largely myogenic. The inability to maintain muscle force due to significantly low levels of PKC activity results in reduced bladder emptying, increased residual volumes, and an overall shortening of the micturition cycle leading to voiding frequency [22].

Partial bladder outlet obstruction, well known for its prevalence in aging males and often secondary to benign prostatic hyperplasia, has also been shown to alter structural proteins within the bladder wall leading to reduced compliance, DO, and frequency of urination. Among the structural proteins altered in PBOO are smooth muscle myosin (SMM) [84] collagen [85], caldesmon [80] and connective tissue [86]. A change in the ratio and organization of smooth muscle to non-smooth muscle elements is generally believed to lead to stiffening of the bladder wall, and a reduction in compliance resulting in a reduced storage capacity, and frequency of urination [77, 80, 85, 87]. However, significant changes in structural elements such as connective tissue, and collagen may represent some of more severe consequences of acute bladder outlet obstruction, associated with bladder decompensation, and may not represent some of the more subtle changes in bladder function that occur due to changes in regulatory proteins and associated with bladder compensation [3, 25, 88, 89].

**Fig. 3:**
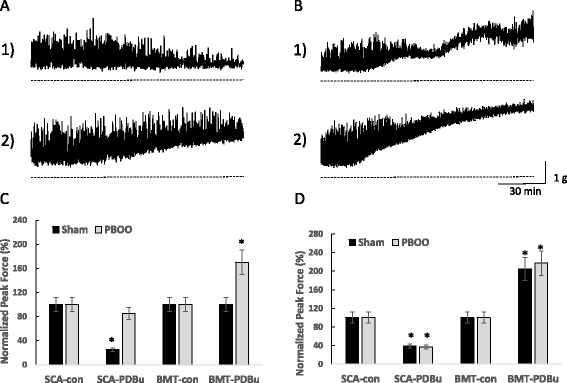
Low and high levels of PKC stimulation by PDBu differentially affect DSM tone and spontaneous contractions. **a** Representative isolated muscle strips showing the effect of low (20nM) PDBu stimulation on sham (*upper trace*) and PBOO (*lower trace*) DSM strips from rabbits. Summary data is presented in **c. b** Representative isolated muscle strips showing the effect of high (100 nM) PDBu stimulation on sham (*upper trace*) and PBOO (*lower trace*). Summary data is presented in **d**. Low PDBu stimulation caused a significant inhibition of spontaneous contraction amplitude (SCA) of the sham muscle strips but only a minor effect in PBOO strips, however low PDBu caused a significant increase in basal DSM tone in the PBOO strips. High PDBu stimulation caused a significant increase in basal DSM tone for both sham and PBOO, however, there was a qualitative difference in the profile of the contractions. The force increase in the sham bladders displayed a biphasic effect whereas an increase in PBOO muscle strips tended to be more linear. High PDBu also significantly reduced the amplitude of spontaneous contractions for both sham and PBOO muscle strips

Figure 3 highlights the changes which characterize a milder form of dysfunction in compensated bladders such as decreased relaxation and increased DSM tone in response to the PKC activator, PDBu. Low levels of PKC stimulation which induce inhibition in normal DSM (panel A1) resulted in increased force in compensated DSM (panel A2). Additionally, moderate to high levels of PKC stimulation caused a modulated rise in tension in normal bladder DSM (panel B1), but a linear increase in the compensated DSM (panel B2). A failure to relax sufficiently and a linear, instead of a modular, increase in DSM tone are the contributing factors to a higher bladder pressure and bladder dysfunction observed in compensated bladders in comparison with normal and sham-operated controls.

## Study limitations

We acknowledge that our study has several limitations. First, we were not able to find any direct data on the role of PKC signaling in detrusor underactivity nor whether PKC pathways could be pharmacologically targeted to improve this condition. Second, we could not find sufficient information on the role of PKC pathways in sensory and efferent innervation of the lower urinary tract. This could be especially important for understanding the role of PKC in other pathological urologic conditions such as chronic pelvic pain (CPP) and bladder pain syndrome (BPS) [90, 91]. For example, a high level of PKC expression and/or activity has been suggested to play a role in feline interstitial cystitis [73], a naturally occurring bladder pain and dysfunctional voiding in cats. It would be of interest to determine whether or not PKC is overexpressed in these bladders, since high PKC stimulation was shown to increase bladder wall tension, and enhance neuronal sensitivity and contractility [8, 22, 25, 28]. Third, future studies focused on the role of PKC in the bladder urothelium are required to clarify the role of epithelial PKC in micturition.

## Conclusions

PKC function in the urinary bladder extends far beyond its contribution, or the lack thereof, to maximum force generation in response to agonists, either directly, or in response to EFS. Data indicate that PKC is involved in the regulation of normal bladder function, and that PKC dysfunction is associated with DO, reduced contractility, and decreased void volume. Relaxation and modulation of spontaneous myogenic, and NVC via PKC-dependent activation of BK and K_ATP_ channels may also be one of its primary functions in support of bladder storage. Both *in vitro*, and *in vivo* studies reveal that PKC may be involved in regulating neuronal activity at the peripheral level in a concentration-dependent manner that may function in a complementary way to facilitate voiding. Finally, the demonstration that PKC dysfunction coexists with pathological changes such as PBOO, DO and is associated with reduced contractility and bladder emptying is further evidence of a significant and important role for PKC in the regulation of urinary bladder function.

## Abbreviations

Bim-1: Bisindilylmaleimide1
BK: Large conductance calcium-activated potassium channel
BSM: Bladder smooth muscle
BPS: Bladder pain syndrome
Ca^++^: Calcium
CPP: Chronic pelvic pain
CNS: Central nervous system
DSM: Detrusor smooth muscle
DO: Detrusor overactivity
EFS: Electrical field stimulation
IF: Integral force
M_3_: Muscarinic
MLCP: Myosin light chain phosphorylation
NVC: Non-voiding contractions
OAB: Overactive bladder
PBOO: Partial bladder outlet obstruction
PDBu: Phorbol-12,13-dibutyrate
PF: Peak force
PLC: Phospholipase C
PMA: Phorbol-12,13-myristate
PKC: Protein kinase C
ROK: Rho-associated kinase
VGCC: Voltage gated calcium channels
SK: Small conductance ca^++^-activated potassium channels
SMM: Smooth muscle myosin
THR: Threonine
